# Spatiotemporal brain dynamics supporting the immediate automatization of inhibitory control by implementation intentions

**DOI:** 10.1038/s41598-017-10832-x

**Published:** 2017-09-07

**Authors:** Michael De Pretto, Lucien Rochat, Lucas Spierer

**Affiliations:** 1 Neurology Unit, Medicine Department, Faculty of Sciences, University of Fribourg, Fribourg, Switzerland; 2 Cognitive Psychopathology and Neuropsychology Unit, University of Geneva, Geneva, Switzerland; 3 Swiss Centre for Affective Sciences, University of Geneva, Geneva, Switzerland

## Abstract

While cognitive interventions aiming at reinforcing intentional executive control of unwanted response showed only modest effects on impulse control disorders, the establishment of fast automatic, stimulus-driven inhibition of responses to specific events with implementation intention self-regulation strategies has proven to be an effective remediation approach. However, the neurocognitive mechanisms underlying implementation intentions remain largely unresolved. We addressed this question by comparing electrical neuroimaging analyses of event-related potentials recorded during a Go/NoGo task between groups of healthy participants receiving either standard or implementation intentions instructions on the inhibition stimuli. Inhibition performance improvements with implementation intentions were associated with a Group by Stimulus interaction 200–250 ms post-stimulus onset driven by a selective decrease in response to the inhibition stimuli within the left superior temporal gyrus, the right precuneus and the right temporo-parietal junction. We further observed that the implementation intentions group showed already at the beginning of the task the pattern of task-related functional activity reached after practice in the group having received standard instructions. We interpret our results in terms of an immediate establishment of an automatic, bottom-up form of inhibitory control by implementation intentions, supported by stimulus-driven retrieval of verbally encoded stimulus-response mapping rules, which in turn triggered inhibitory processes.

## Introduction

Referred to as ‘inhibitory control’, the ability to suppress ongoing or planned cognitive or motor processes is a key executive function mainly involved in overriding impulsive or habitual responses^1^. Current models of the functional architecture of inhibitory control indicate that response inhibition consists in the following multi-phase process: after an initial perceptual discrimination of the stimuli associated with execution vs inhibition goals within sensory and associative cortices around 50–150 ms post-stimulus onset, whether inhibition must be engaged is determined based on the retrieval of stimulus-response mapping rules in memory at ca. 100–250 ms by parietal areas^2, 3^. Then, at 250–300 ms, when stop stimuli are identified and action cancellation decided, response inhibition is initiated within right inferior frontal a gyrus (rIFG) and preSMA to eventually stop motor activity via projections to the basal ganglia and thalamus^4–6^.

Based on evidence for a key role of inhibitory control deficits in the emergence and maintenance of impulse-control disorders such as addiction or bulimia^7–9^, several studies have tested whether inhibitory control training might help improving the control of impulses. They revealed that reinforcing top-down control had only modest effect^10^, most likely because these interventions did not reduce the voluntary effort required to initiate executive processes^11^. To address this issue, some authors advanced that cognitive interventions for improving impulse control should rather aim at developing automatic forms of response inhibition that require less conscious efforts to be engaged (see ref. 11 for a review).

Automatizations of inhibitory control have been observed in studies involving the practice of Go/NoGo tasks in which a given ‘NoGo’ stimulus was repeatedly associated with stopping goals^12, 13^. After such training regimens, inhibitory control became directly triggered by the stimuli via the brain regions implementing stimulus-response (S-R) mapping rules (parietal cortices at 100 ms)^14^. As a result, response inhibition processes bypassed the slow, intentional engagement of inhibition process, which in turn improved performance. However, since ‘tagging’ a given stimulus with inhibition goals required many repetitions, establishing new associations between a stimulus and an inhibition based on task practice is time consuming.

Self-regulation strategies such as “Implementation intentions” (II) constitute an interesting approach to circumvent this problem: within II procedures, stimulus and responses are rapidly associated by formulating an “If-Then” plan (“If I encounter situation X, then I produce behavior Y”)^15, 16^. In an inhibitory control Go/NoGo task, the instructions would for example be “If I see a red square, then I withhold my response”. In such plans, the “if” component specifies a suitable occasion for the implementation, whereas the “then” component identifies an adequate response to these target events (i.e. a behavior allowing achieving expected outcomes). By creating a link between the situation and the expected behavior, II enable an automatic initiation of adequate behaviors^15–19^.

While II strategies have proven successful in improving inhibitory control performance^17, 18, 20–22^, only few studies focused on their underlying neural mechanisms^23–25^. Paul and colleagues^24^ showed that II decreased the amplitude of the P300 event-related potential (ERP) component in healthy controls during a Go/NoGo task and correlated positively with the percentage of correctly inhibited responses in an ADHD clinical population. The authors localized the source of the P300 peak within the anterior cingulate cortex, and in line with the ‘automatization’ hypothesis, they interpreted their findings in terms of II improving performance by reducing the efforts required to engage inhibition processes. In another EEG study examining the influence of II on emotion regulation, II were shown to modulate the early latency P100 ERP component, suggesting that they improved performance by biasing attention toward the stimuli involved in the II^25^. In a similar study, Hallam *et al*.^23^ further reported reductions in fMRI activity in within the left amygdala, and increased activity in the right IFG and the ventro-parietal cortex by II. The authors interpreted these results as indicating an improvement of the processing of the stimuli and the access to the expected response scheme by II. Of note, functional studies of II further showed that they modulated activity within the superior temporal gyrus and precuneus areas^23, 26^. The involvement of these areas in auditory and self-centered mental imagery suggests that II favored the retrieval of verbally encoded response schemes^27–31^.

While the literature reviewed above provides information on the putative neurocognitive mechanisms supporting the effect of II on inhibitory control, they did not disentangle the precise spatio-temporal brain dynamics underlying II.

To address this question, the current study capitalized on the high temporal and spatial resolution of electrical neuroimaging approach^32, 33^; we compared the event-related potentials and source estimations to Go and NoGo stimuli recorded during a classical Go/NoGo task between two groups of healthy participants who received either standard instructions or instructions formulated as implementation intentions. At the behavioral level, we hypothesized that as compared to standard instructions, II instructions would improve inhibition performance. At the electrophysiological level, because the formulation of the II focused on the NoGo stimuli, we expected that they would selectively modulate inhibition (but not execution) processes during decisional processing phases corresponding to the memory retrieval of stimulus-response mapping rules and response selection (i.e., at 100–250 ms post-stimulus onset), and within areas involved in the interfacing between stimulus and response, and auditory/verbal imagery (i.e. a decrease in superior temporal areas reflecting a more efficient encoding of the instruction^23, 26^).

Finally, since II have been considered as involving an associative learning mechanism putatively corresponding to those manifesting with task practice, we further examined whether and how II influenced task learning by comparing between the two groups of participants the ERPs to the NoGo stimuli between the beginning and the end of the task. We predicted that practice-related automatization in the control group receiving standard instructions would manifest as functional changes corresponding to those associated with II, i.e. an increase in parietal activity^14^ and a decrease in fronto-temporal activity^23, 26^.

## Results

### Behavioral results

Table 1 reports the group means and the results of the analyses on the response time to Go trials (RT) and on the false alarms to NoGo trials (FA rate) for each experimental condition. Benchmark for effect size interpretation is by Cohen, 1977 (D of 0.2 = small; 0.5 = medium; 0.8 = large). Table 2 reports the results of the questionnaires analyses.

**Table 2:** Scores and statistical analyses of the questionnaires.

	Mean scores (SD)		SI vs II t-tests (t_30_; p-value; Cohen’s d)
	SI	II	
Urgency (UPPS-P)	9.7 (1.9)	9.6 (2.0)	0.16; 0.876; 0.05
Emotional arousal (PANAS)	48.1 (5.7)	47.5 (9.7)	0.21; 0.834; 0.08
Anxiety (STAI)	39.2 (4.8)	39.1 (9.4)	0.05; 0.959; 0.01

Manipulation check data showed that 67% SI participants and 94% II participants mentally repeated the instruction. In the II group, 94% of the participants used a strategy focusing on the stimuli to inhibit; 65% of the participants reported repeating the instruction in the if-then form, and the remaining simply repeated the stimulus to inhibit (for example repeating “blue”). In that group, one participant reported focusing on the Go stimuli (mentally representing the Go stimuli). In the SI group, 40% of the participants focused on the stimulus to inhibit (for example repeating “blue”) and 27% reported focusing attention on the Go stimuli. No participant in the SI group reported using an If-Then formulation. The 33% remaining participants did not report using a particular strategy. These results thus confirm that: (i) the two groups indeed differed in their strategies (most of the participants in the II groups used the If-Then strategy and none in the SI group); and ii) II formulation dramatically increased the utilization of a specific (II) strategy, thereby reducing the variability in response strategy. Consequently, although the instructions between SI and II groups may look quite similar, the difference of performances could be accounted for by the instructional manipulation. Indeed, by explicitly forming an implementation intention in the II group, the mental representation of the critical situation became highly activated and hence more accessible, which purportedly improved inhibitory control.

#### Immediate effect of task instructions (Group SI vs Group II)

**Figure 1:**
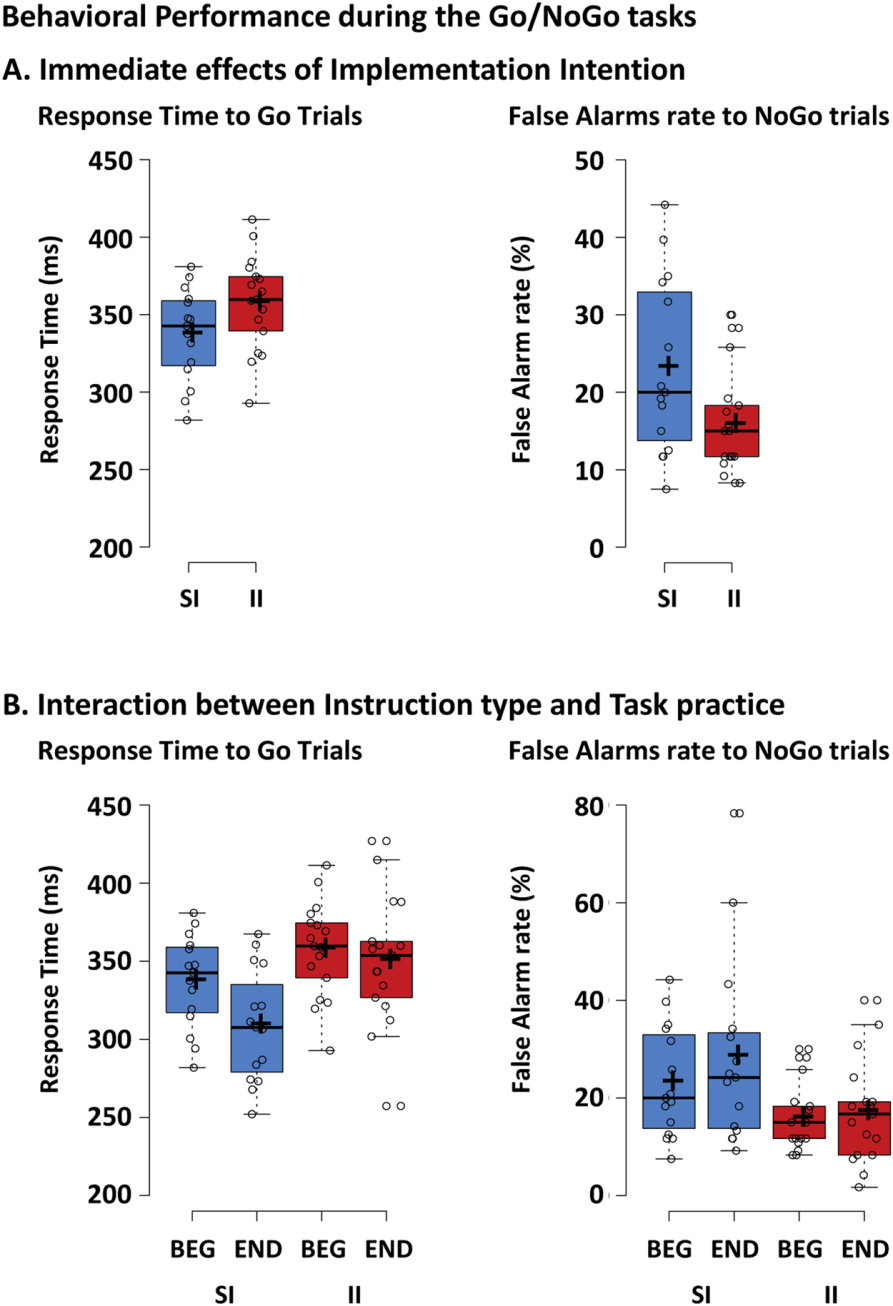
Behavioral performance during the Go/NoGo tasks. (**A**) The performance was better in the implementation intentions (II) than in the standard instructions group (SI), as indexed by less false alarms to NoGo stimuli. (**B**) With practice, response times to Go stimuli decreased in the SI group, whereas false alarms rate to NoGo stimuli remained stable in each group. Individual subject’s data points, the median (horizontal line), the mean (cross), and confidence intervals (Tukey whiskers) are represented. SI: control group receiving standard instructions; II implementation intentions group; BEG: beginning of the session; END: end of the session.

The unpaired t-tests showed a trend for slower RTs in the II than SI group, and a lower FA rate in the II than SI group with a large effect size (Fig. 1A, Table 1 for the p-values and effect sizes). This pattern was confirmed by the analysis of the sensitivity and response criterion, which showed that the II group had a largely more conservative response strategy than the SI group (more negative C value).

**Table 1:** Scores and statistical analyses of the behavioral performance during the Go/NoGo tasks.

		Mean (SD)		Immediate effect	Practice effects		
		SI	II	SI vs II (t_30_; p-value; Cohen’s d)	Group (SI; II) (F_1,30_; p-value; ηp^2^)	Session (BEG; END) (F_1,30_; p-value; ηp^2^)	Group x Session (F_1,30_; p-value; ηp^2^)
Response Time (ms)	BEG	337.1 (29.9)	357.5 (30.6)	−1.90; 0.068; 0.70	7.65; 0.010; 0.20	10.83; 0.003; 0.27	3.75; 0.062; 0.11
	END	308.9 (36.1)	350.1 (41.5)				
False Alarms Rate (%)	BEG	23.1 (11.3)	15.8 (6.8)	2.26; 0.031; 0.82	5.39; 0.027; 0.15	2.83; 0.103; 0.09	1.02; 0.321; 0.03
	END	28.4 (19.4)	17.1 (10.6)				
D prime	BEG	2.8 (0.6)	3.0 (0.5)	−1.37; 0.181; 0.50	3.16; 0.086; 0.10	3.95; 0.056; 0.12	0.90; 0.351; 0.03
	END	2.4 (0.9)	2.9 (0.8)				
C Criterion	BEG	−0.6 (0.2)	−0.5 (0.1)	−2.25; 0.032; 0.82	5.32; 0.028; 0.15	1.46; 0.237; 0.05	0.22; 0.646; 0.01
	END	−0.6 (0.3)	−0.4 (0.3)				

Note. SI = control group receiving standard instructions and II = implementation intentions group. We tested for normality of all variables using a Kolmogorov-Smirnov test, which indicated that the data were normally distributed in both groups and for all conditions.

#### Effect of task instructions on the changes in performance with practice (Group x Session interaction)

The Group (SI; II) x Session (BEG; END) mixed ANOVA on RT (Fig. 1B) revealed a small main effect of Group driven by shorter response time in the SI than II group, and a small main effect of Session driven by shorter response time at the end than at the beginning of the session (see Table 1 for the p-values and effect sizes). The interaction term was only marginally significant, and post-hoc t-tests revealed a decrease in response time with practice in the SI group (p = 0.002), but not in the II group (p = 0.358). The ANOVA on FA rate revealed only a main effect of Group driven by lower FA rate in the II than SI group.

Of note, tests for Levene’s homogeneity of variance indicated unequal variance for the FA rate at the beginning of the session between the II and SI groups (F(1,30) = 6.41, p = 0.017). We thus corrected the unpaired t-test on FA rate using a t-test with a Satterthwaite approximation for the degrees of freedom. After correction, the t-test still indicated the II vs SI group difference (t(1,22.24) = 2.19, p = 0.039).

A direct analysis of the speed-accuracy tradeoff (SAT) revealed significant negative correlations between the RT and the FA at the end but not at the beginning of the training for both the SI and II group (SI BEG r = −0.536; p = 0.040; SI END r = −0.607*; p = 0.016; II BEG r = −0.064; p = 0.806; SI END r = −0.767*; p = 0.000; * = significant correlation with Holmes-Bonferroni corrected α threshold).

### Electrophysiological results

#### Event-related potentials and electrical source estimations

**Figure 2:**
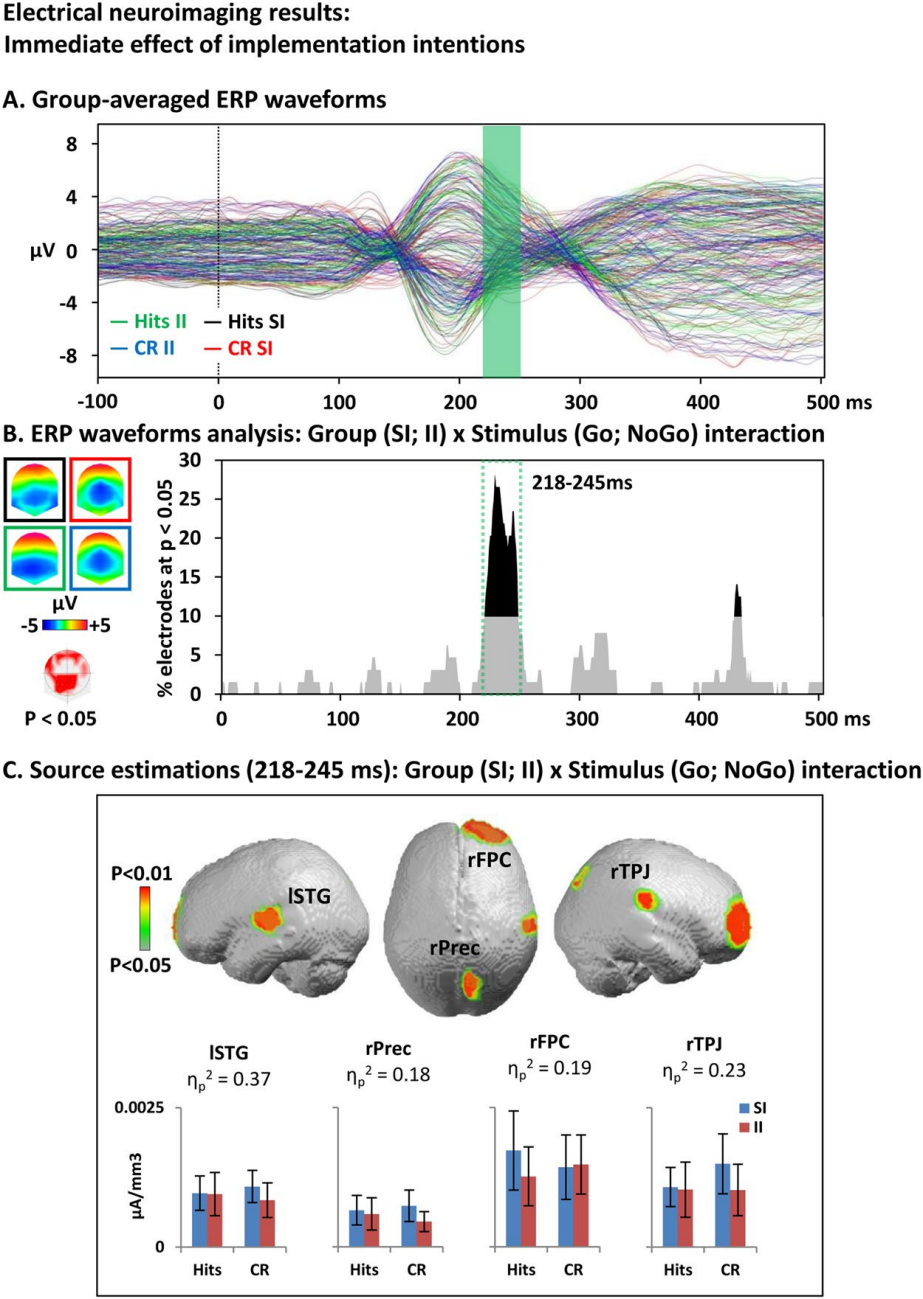
Electrical neuroimaging analysis of the immediate effect of implementation intentions. (**A**) Group-averaged event-related potentials (ERP) for hits to Go trials in the standard instructions (SI) group (black line) and implementation intentions (II) group (green) and for correct rejections to NoGo in the SI group (red) and the II group (blue). (**B**) Electrode-wise statistical analyses of the ERPs. The graph depicts for each post-stimulus time point the percentage of electrodes showing a significant Group x Stimulus interaction (p < 0.05). The 218–245 ms period showed a sustained interaction effect (> 10 ms for > 10% of the electrodes; green dotted square). The topographic map (nasion upward) represents in red the electrode sites showing a significant interaction, and on the left the topographies of the ERPs for all groups and conditions (same colors as in A). (**C**) Statistical analysis of the distributed electrical source estimations over the period defined in (**B**). (p < 0.05; K_E_ = 15). SI: control group receiving standard instructions; II implementation intentions group; CR: correct rejection; K_E_: spatial extent criterion.

Immediate effect of task instructions (Group x Stimulus interaction). Group-averaged ERP waveforms are depicted to help assess the quality of the signal (Fig. 2A).

The investigation of the immediate effect of implementation intentions (II) vs standard instructions (SI) on the ERP to HIT vs CR trials was based on the analysis of the Group (SI; II) x Stimulus (HIT; CR) interaction at each time frame across the whole electrode montage: There was one period of sustained interaction (p < 0.05; >10% electrodes) from 218 to 245 ms, mostly over centro-occipital electrode sites (Fig. 2B).

Statistical analyses of sources estimations over the periods of interest identified with the ERP analyses revealed significant (p < 0.05; k_E_ ≥ 15) Group x Stimulus interaction in the right precuneus (MNI: 9 -68 38), temporoparietal junction (59 -22 25), and anterior frontopolar cortex (MNI: 23 67 4); and the left insula/superior temporal gyrus (MNI: -45 -10 7; Fig. 2C). Plots of the group-averaged current source density values indicated that the interaction was driven by a reduced activity of the anterior frontopolar cortex for the II vs SI group during successful Go trials (HIT) and equivalent activity during successful NoGo trials (CR). For all the other brain areas, activity was equivalent during hits and reduced for the II vs SI group during CR.

#### Effect of task instructions on the changes in the ERP to the NoGo stimuli with practice (Group x Session interaction)

**Figure 3:**
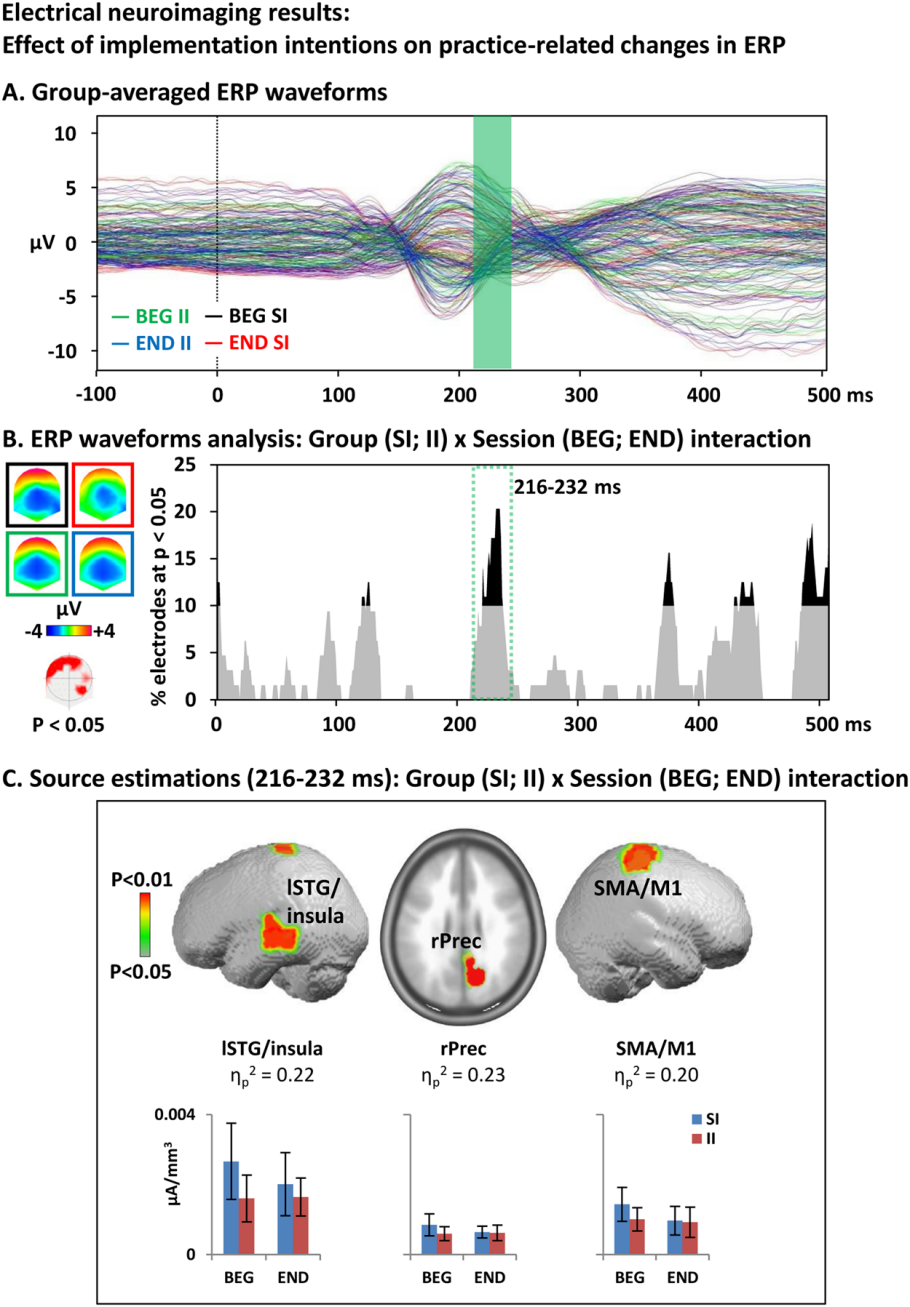
Electrical neuroimaging results: Effects of implementation intentions on practice-related ERP modulations. (**A**) Group-averaged event-related potential (ERP) for correct rejection to NoGo trials at the beginning of the session in the standard instruction (SI) group (black trace) and implementation intentions (II) group (green) and at the end of the session in the SI group (red) and the II group (blue). (**B**) Electrode-wise statistical analyses of the ERPs. The graph depicts for each post-stimulus time point the percentage of electrodes showing a significant Group x Session interaction (p < 0.05). The 216–232 ms period showed a sustained interaction effect (>10ms for > 10% of the electrodes; green dotted square). The topographic map (nasion upward) represents in red the electrode sites showing a significant interaction, and on the left the ERP topography for all groups and conditions (same colors as in A). (C). Statistical analysis of the distributed electrical source estimations over the period defined in B. (p < 0.05; K_E_ = 15). SI: control group receiving standard instructions; II implementation intentions group; BEG: beginning of the session; END: end of the session; K_E_: spatial extent criterion.

Group-averaged ERP waveforms are depicted to help assess the quality of the signal (Fig. 3A).

The investigation of the effect of II vs SI instructions on changes in the ERP to the NoGo stimuli with task practice was based on the analysis of the Group (SI; II) x Session (BEG; END) interaction at each time frame across the whole electrode montage on CR trials. There was one period of sustained interaction (p < 0.05; >10% electrodes) from 216 to 232 ms, mostly over frontal electrode sites (Fig. 3B). There were signs of late latency interactions (400–500 ms) but that did not meet our duration criterion.

The statistical analyses of sources estimations revealed significant (p < 0.05; k_E_ ≥15) Group x Session interaction in the right precuneus (MNI: 9 -67 30), SMA/M1 (MNI: 31 -21 73), and the left middle/superior temporal gyri (MNI: -51 -15 7; Fig. 3C). Plots of the current density values indicated that the interaction was driven by a reduced activity in all these brain areas for the II vs SI group during the BEG blocks. After practice (END), the activity in the SI group had decreased to reach a level comparable to those of the II group.

We further calculated correlations between the activity (current densities) of the clusters showing the significant interactions and the questionnaires on urgency and arousal state but no result reached Bonferroni corrected p-value (p = 0.0015).

## Discussion

Implementation intentions, the formulation of task instructions as “If-then” plans, improved inhibitory control performance and were associated with ERP modulations at 200–250 ms post-stimulus onset within fronto-temporal areas. We interpret these results in terms of II facilitating the retrieval of verbally encoded stimulus-response mapping rules and the automatization of the triggering of inhibition processes by the NoGo stimuli. A second analysis of the effect of task practice in the group receiving II vs standard instructions supported this ‘automatization’ account of the effect of II by showing that such formulations of the instructions resulted in the immediate installation of task-related patterns of brain activity corresponding to those observed after a practice when standards instructions were given.

As compared to the group receiving standard instructions (SI), II associating NoGo stimuli to inhibition goals resulted in fewer commission errors (false alarms) and in a more negative C Criterion, which corroborates previous studies stressing the efficacy of II to improve self-control (see e.g. ref. 22). This improvement in inhibition accuracy was, however, accompanied by a tendency for slower response time. While this pattern suggests a speed-accuracy tradeoff, our specific SAT analysis indicated no relationship between the RT and the FA rate.

Yet, together with the signal detection theory analyses, the effect of II on FA but not RT suggests that the II resulted in a more conservative response strategy. Since in our task, response speed was required via standard instructions, the decrease in FA but not RT in the II group suggests that II had stronger effects on behavior than standard instructions. The SI on responding as fast as possible to Go stimuli and the feedback on response speed systematically presented to participants after Go trials during our task were not sufficient to balance the effect of II on response accuracy (for similar findings see refs 21 and 24). Such pattern for a selective effect of SI on FA when only this aspect was the focus of II was also observed in the reverse direction, with increased in FA rate when II focused on response speed only^17, 18^.

The electrophysiological analyses revealed an effect of II task instructions on the ERP to the Go vs the NoGo stimuli at 200–250 ms post-stimulus onset, driven by a decrease in the responses of right frontopolar cortices (FPC) to Go trials and of right precuneus and temporoparietal junction (TPJ) as well as the left superior temporal gyrus (STG) to NoGo trials. In line with previous reports on the functional effects of II formulated verbally^23, 26^, we interpret this effect as reflecting a shift to more automatized, bottom-up, stimulus-driven forms of inhibitory control in which inhibition processes were directly triggered by the reactivation of verbally encoded stimulus-response mapping rules by the NoGo stimuli.

This interpretation is first supported by the 200–250 ms latency of the Group (SI; II) x Stimuli (Go; NoGo) interaction. This latency indeed corresponds to beginning of the N2/P3 complex^34, 35^, a process taking place between the retrieval of S-R mapping rules and the implementation of inhibitory processes during Go/NoGo tasks^36^. Interestingly, the electrophysiological activity over this time period has been found to correlate with inhibition accuracy during motor inhibitory control^37^, further supporting the functional relevance of this processing step for inhibition performance. Similar associative learning mechanisms have been advanced as supporting inhibitory control performance improvement with practice^12^. However, when the associations between NoGo stimuli and NoGo goals are induced only by task practice, ERP modulations manifest around 100 ms post stimulus onset, and within parietal areas^14^. The later latency of stimulus-response associations induced by II than by repeated associations between the NoGo stimuli presentation and the inhibition process as in Manuel *et al*.^14^ likely reflects that with II, the triggering of inhibition processes by the NoGo stimuli involved an additional phase of reactivation of verbally encoded instructions.

The variations in the latency of the effect of II observed when emotional stimuli or a complex task are used suggest that the neurocognitive mechanisms underlying II vary depending on the experimental paradigm. II during an emotional regulation tasks was for example associated with modulations during the P100 ERP component, suggesting that II improved the preattentive regulation of emotionally laden stimuli^25^. Paul *et al*.^24^ reported change in the P300 component during a slow and cued inhibitory task and suggested that II modulated decision making processes. While the difference in the type of analyses conducted in our vs previous studies prevents direct comparisons across studies, the effects of II generally manifested 100–300 ms post-stimulus onset, which corresponds to decisional processing phases taking place between perceptual discrimination and task-specific processes.

The involvement of the insula/STG and precuneus in II supports that this self-regulation strategy influences stimulus-responses mapping processes. These regions have indeed been involved in auditory imagery/internal speech^27, 29–31^ and in attention shifting, respectively (see ref. 28 for a review). While both groups had to rely on the retrieval of verbally encoded response schemes to perform the task, the decrease in insula/STG and precuneus in the II group suggests that II facilitated this process. Decreases in activity within task-related areas have indeed repeatedly been associated with practice-induced task automatization, especially in executive tasks (e.g. see refs 38–41). Further supporting this interpretation, the effect of the II on the insula/STG and precuneus were specific to the NoGo condition on which the II instructions were focused.

In the NoGo condition, we further found that compared to SI, the II instructions were associated with a decrease in rTPJ activity, a region involved in facilitating the interaction between goal-directed and stimulus-driven attention (see refs 42–44 for a review). Accordingly, II may have strengthened the associations between the NoGo stimuli and the inhibition goals, thereby facilitating a shift from a deliberate, intentional control of inhibition processes to a more bottom-up, stimulus-driven form of inhibition.

The hypothesis for a shift to stimulus-driven control of response with II is also supported by our result for a specific decrease in responses to the Go trials within the right FPC in the II compared to SI group. Indeed, Gilbert *et al*.^26^ reported a sensitivity of these regions to conflicts between self-generated vs externally triggered response schemes. Here, because II instructions focused on responses to NoGo only, the reduced activity of this area might indicate a more balanced integration of internal and external information with II even during Go trials, though with a cost on response time.

Task automatization based on learning associations between NoGo stimuli and inhibition goals has been previously advanced as a putative mechanism of inhibitory control improvement with practice^12–14^. Since II have been advanced to be supported by a corresponding mechanism, we tested the prediction that the immediate effects of II would correspond to those induced by practice in the SI group.

Behaviorally, we found a tendency for a Group (SI; II) x Session (BEG; END) interaction effect on RT but not on FA. While in the SI group, we replicated previous findings on the effect of Go/NoGo training with a decrease of RT associated with stable FA rate during the task^45, 46^, we observed no change of RT or FA rate with practice in the II group. Implementation intentions thus immediately improved inhibitory control accuracy (FA rate) to a level preventing -or at least limiting- any further improvement at the level of RT. Indeed, by putting a strong pressure/emphasis on FA, the II actually prevented any change in RT because for a given maximal IC capacity, an improvement in RT could only be achieved at the expense of FA.

We would further note that aside from automatization, non-specific practice effects such as learning to find an appropriate balance between speed and accuracy (here by prioritizing speed over accuracy) might also have contributed to the observed effects.

The comparison between the ERPs to NoGo trials at the beginning vs. the end of the practice in the II vs SI group revealed an interaction at a latency corresponding to that of the immediate effect of II, at 200–250 ms. The Group x Session interaction was driven by larger decreases in activity in the SI than II group within bilateral SMA/M1 cortices, left middle/superior temporal gyri, and right precuneus. With practice, the SI group showed a decrease in activity within these areas up to a level corresponding to those observed in the II group, which did not show any functional change with practice. This pattern of results supports the ‘automatization hypothesis’ advanced to account for the immediate effect of II: with practice, the SI group shifted from a controlled to automatic response mode corresponding to the mechanism immediately available to the II group via the instructions they received. This interpretation is in line with a recent proposition by Verbruggen *et al*.^13^according to which implementation intentions may lead to a “prepared reflex”. Indeed, the linking of critical situations or cues to specific actions (as is the case in an If-Then contingency) may stimulate proactive allocation of attention, increased monitoring for cues and response preparation, so that goal-directed actions would not require much control anymore or a conscious intent (see refs 15 and 19). Thus, with II, inhibitory control becomes (at least partly) directly triggered by information in the environment.

Finally, the effect in SMA/M1 putatively reflects reduced movement preparation, resulting in more efficient inhibition to NoGo trials. In addition to its role in motor preparation^47, 48^, the SMA has also been associated with imagining the production of movements^49, 50^. More specifically, Lima *et al*.^51^ suggested that the pre-SMA/SMA was involved in auditory perception or imagery tasks to modulate motor response based on previous sensorimotor experience. The role of the SMA in motor and auditory imagery might explain why only this area of the cortico-basal network controlling inhibition showed differential practice effects between the two groups.

The present study suffers the following limitations: First, we did not assess whether the behavioral and neurophysiological effect of implementation intentions persist in time. Previous literature on this question suggests that compared to control conditions, the effect of II on specific behaviors (e.g., eating balanced meals) may persist over long-time period (see e.g. ref. 52), although the durability of II efficacy has not been specifically examined on response inhibition. Further limiting the clinical relevance of our study, we don’t know if the present result could be generalized to clinical population. One could indeed advance that because of the different neurocognitive ‘context’ of clinical populations, II would have different effects and neural correlates as in the healthy population tested here. However, most of previous studies on II conducted on clinical populations showed larger effects in clinical than control groups. Another limitation is that we only tested the effect of verbally presented II, and whether corresponding effects would be observed with visually presented instructions remains unclear. Finally, our interpretations partly rely on inferring the cognitive processes modified by the implementation intentions based on the brain regions showing changes in activity across groups. The reliability of such reverse inferences is particularly limited when activity in brain regions with a low functional specificity is interpreted^53^.

## Conclusion

Our collective results suggest that implementation intentions improve inhibition control by facilitating the retrieval of verbally encoded stimulus-response mapping rules during the inhibitory control decisional processing phase immediately preceding the implementation of the inhibition process. These data also support that implementation intentions automatize response control, with its effect mimicking those induced by short practice.

## Methods

### Participants

Thirty-six right-handed adults participated in the study. Our sample size was calculated based on previous ERP literature on inhibitory control training (e.g. refs 14, 24, 25, 45 and 54) reporting medium to large effect size; for a power of 0.8 to detect effect of d = 0.8 or f = 0.3–0.4 for α = 0.5 with one-tailed independent-sample t-tests or a within-between subject interaction, a sample of n = 15–20 was necessary. However, since our power calculation was mostly based on within-subject training effects, the present study may be underpowered to detect between-subjects instructional main effects if the effect sizes are less than d = 0.9. Participants were recruited with flyers on the University campus and received a financial compensation for their participation. All had normal or corrected to normal vision, and no history of neurological or psychiatric illness. Informed consent was obtained for all participants. The methods were carried out in accordance with the relevant guidelines and regulations. All experimental protocols were approved by the “Commission cantonale d'éthique de la recherche sur l'être humain (CER-VD)” of Lausanne, Vaud, Switzerland. Four subjects were excluded from the analyses: two did not follow the instructions (one inhibited responses to the false NoGo stimuli in the first block, and one reported trying to guess the nature of the upcoming stimuli); one received the wrong block for blocks three and four; one showed noisy EEG signal. Of the thirty-two remaining participants, fifteen (7 females, 8 men; 22.3 ± 2.3 years) received the standard instructions and seventeen (8 females, 9 men; 22.7 ± 1.7 years) received the implementation intentions instructions. The participants were randomly assigned to the experimental groups.

### Stimuli and task

The task was adapted from Hartmann *et al*.^45^; the participants performed a visual Go/NoGo task for which only the instructions differed between the control and the implementation intentions groups. Stimuli consisted of three different letters (A, E, O) in three different colors (blue, green, red) presented at the center of a black background. The possible combination of letters and colors yielded nine different stimuli. Using a letter-color compound for the stimuli enabled generating many different stimuli (and thus to vary them systematically throughout the session) while keeping the different stimuli easily discriminable. In turn, performance improvement with practice could not be attributable to learning to discriminate between the stimuli. In each block, NoGo stimuli were either all letters of a given color or all colors of a given letter. All the remaining stimuli were used as the Go stimuli. There was thus a total of six different blocks, each with one of the six different NoGo stimuli. Participants had to press as quickly as possible with their right index finger on a response box to Go stimuli and to withhold their responses to NoGo stimuli.

### Procedure

The procedure was the same as in Hartmann *et al*.^45^; participants were seated in front of the computer screen in a sound-attenuated booth. Stimulus presentation and response recording were controlled by the E-Prime 2.0 software (Psychology Software Tools, Inc., Sharpsburg, PA).

The Go/NoGo task was split in 12 blocks of 60 trials (30 Go and 30 NoGo presented randomly) separated by 2 min breaks. Each block (i.e. with a given NoGo stimulus) was presented twice in a row, with NoGo being specific letters independently of their color for two blocks, then specific colors independently of the letter for two blocks, etc. The order of the block pairs was different for each participant. The stimulus-response mapping rule was varied every two blocks to limit the automatization of the inhibition process that typically takes place when the Go and NoGo stimuli are kept constant in Go/NoGo tasks (see refs 12 and 55 for discussion).

Before each block, participants were presented with written instructions indicating which stimuli will be the NoGo for the upcoming block. To maintain time pressure, a feedback “too late!” was presented if the RT was above the response time threshold (RTt). The RTt was computed as 90% of the mean RT to the Go stimuli of the preceding block. In the first block, the RTt was set at 380 ms for all participants, corresponding to the mean RT observed in Hartmann *et al*.^45^. This procedure enabled maintaining the same level of time pressure across participants and blocks, i.e., independently on any initial inter-individual differences in Go/NoGo performance and on performance improvement with training (see refs 14 and 56 for similar procedures).

**Figure 4:**
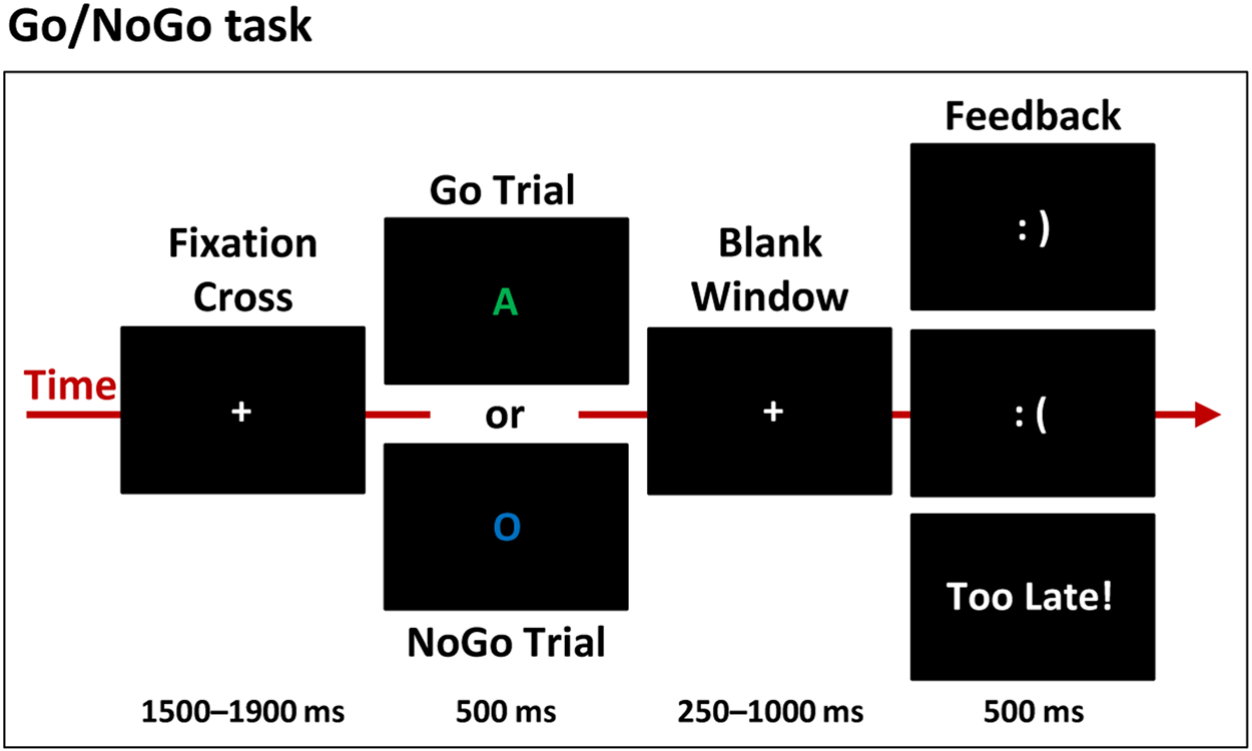
Experimental Go/NoGo paradigm. Participants had to respond as fast as possible to the Go stimuli while withholding their response to the NoGo stimuli. In a given block, NoGo stimuli were either all letters of a given color or all colors of a given letter; Go trials were all the remaining stimuli. A feedback was provided on response speed and accuracy. The control and implementation intentions groups completed the same task except the way instructions were formulated.

Each trial started with the presentation of a grey fixation cross during a time range of 1500 to 1900 ms, followed by the stimuli (500 ms) and a blank period (1000 ms; Fig. 4). The blank period terminated when the participant responded, but had a minimal duration of 250 ms. Participant could respond from the onset of the stimulus to the end of the blank period. The participants then received a feedback on their performance for 500 ms: a happy smiley icon after hits (response after a Go stimulus) and correct rejections (no response after a NoGo stimulus); a “Too late!” feedback for hits with a RT > RTt; and an unhappy smiley after misses (no response after a Go stimulus) and false alarms (response after a NoGo trial). The total length of the session was of ca. 1 hour.

#### Instructions

At the beginning of the recording session, the experimenter read the instructions to the participant. Since the control and the implementation intentions group differed only in terms of how the instructions were given, and to reduce intra-group variability at this level, the experimenter read the instructions rather than explaining the task without written support.

In the control group receiving standard instructions (SI), the experimenter told participants to “press as quickly as possible on the response button as soon as a letter appears. However, sometimes the letter will be [NoGo criteria (a specific letter or color)]. In that case, you have to withhold your response”.

In the implementation intentions (II) group, the experimenter told participants to “press as quickly as possible on the response button as soon as a letter appears. However, sometimes the letter will be [NoGo criteria]. If the letter is [NoGo criteria], then withhold your response”.

Since the NoGo changed systematically, written instructions were again presented before each block, again formulated as standard instructions for the SI group and as if-then plan for the II group.

#### Questionnaires

To control that both groups did not differ at the level of psychological variables known to influence the efficiency of implementation intentions, the participants completed questionnaires of impulsivity, and specifically urgency trait (UPPS Impulsivity Scale^57, 58^), emotional arousal state (the Positive and Negative Affect Schedule, PANAS^59, 60^), and anxiety trait (the State and Trait Anxiety Inventory, STAI^61, 62^). Based on previous studies showing that the influence of urgency and emotional arousal on implementation intentions were independent of the valence, we assessed these dimensions with a score in which the positive and negative scores were collapsed together^20^.

At the end of the session, a debriefing questionnaire was used to assess whether the participants employed a strategy to withhold their response when the target stimulus appeared; mentally repeated the instructions; and imagined themselves doing the task and withholding their response when the target stimulus appeared. The questionnaire used “Yes/No” questions and if “Yes”, the participants were required to explain their answer.

### Behavioral analyses

Inhibitory control performance was evaluated with the mean response time to Go stimuli and the false alarm rate to NoGo stimuli (i.e., the percentage of unsuccessful inhibitions). Trials with RTs differing more than two standard deviations from the participant’s mean RT were discarded from further analyses (mean 4.4% of all the trials). Immediate effects of II were evaluated by averaging the scores of the first four blocks of the task (condition “beginning”, BEG). Effects of practice were evaluated by comparing the average score of the first four blocks to the average scores of the last four blocks (condition “end”, END). Behavioral data were also analyzed according to signal detection theory^63^. Sensitivity (d′) was calculated as: d′ = z(Hit)−z(FA) and C as: C = (z(Hit) + z(FA))/2); where z(Hit) and z(FA) represent the transformation of the Hit and False-Alarm rates into z-scores. For the calculations, we incorporated the automatic correction suggested by Macmillan and Creelman^64^ (p. 14) for cases where observed FAs = 0 or Misses = 0.

Between-group differences during the BEG blocks were assessed with two-tailed unpaired t-tests. Effects of practice were assessed with mixed repeated measure ANOVAs with Groups (SI vs II) as between-subject factor and Sessions (BEG vs END) as within-subject factor. Normality and homogeneity of variance was controlled, and we report corrected statistics when necessary. For all statistical tests on behavioral data, the threshold for significance was set at p < 0.05 and effect sizes are reported.

### EEG recording and preprocessing

Continuous electroencephalogram (EEG) was recorded with a sampling rate of 1024 Hz through a 64-channel 10–20 Biosemi Active-Two system (Biosemi, Amsterdam, Netherlands), referenced to a ground circuitry (common mode sense/driven right leg ground or CMS–DRL, placed on each side of POz). This circuitry consists of a feedback loop driving the average potential across the montage as close as possible to the amplifier zero (cf. the Biosemi website for a diagram; http://www.biosemi.com/pics/zero_ref1_big.gif).

Offline analyses were performed with the Cartool software^65^, and statistical analyses were performed with the STEN toolbox (http://www.unil.ch/line/home/menuinst/about-the-line/software--analysis-tools.html).

Before segmenting the raw EEG into peri-stimulus epochs, the continuous raw EEG data were filtered with a second order Butterworth with −12db/octave roll-off; 0.1 Hz high-pass, 40 Hz low-pass; 50 Hz notch filter, and the events were re-labelled according to their associated behavioral outcome. Only the trials associated with correct responses were included in the ERP (successful Go trials, HIT; and successful NoGo trial, hereafter referred to as correct rejections, CR). For each block, the number of HIT and CR trials was then matched to control for difference in signal-to-noise ratio in the ERPs across conditions: only the first n trials of the condition with the highest number of available trials were further processed, with n being the number of trials available for the condition with the lowest number of available trials.

EEG epochs from 100 ms pre-stimulus to 500 ms post-stimulus onset were averaged across trial for Go HIT and NoGo CR separately for the first four blocks (BEG) and the four last blocks (END) of the task. This choice represented the optimal balance between the signal-to-noise ratio in the ERP (maximizing the number of epoch included in each ERP) and the effects of practice (maximizing the duration of the practice between the BEG and END conditions). Eye blinks and other artefacts were removed by automatically rejecting epochs with at least one time frame at one or more electrodes showing a voltage higher or lower than 80 μV. After epoch averaging, electrodes still showing a bad signal were interpolated using 3D splines^66^ (mean 1% of electrodes interpolated). This procedure eventually resulted in the following number of epochs included in the ERP: for BEG HIT, 83 ± 19 epochs in the SI group and 93 ± 11 epochs in the II group; for BEG CR, 83 ± 14 epochs in the SI group and 93 ± 13 epochs in the II group; for END CR, 78 ± 25 epochs in the SI group and 87 ± 23 epochs in the II group. To control that differences in the number of accepted epochs across conditions did not impact the results of the ERP analyses, we applied to the number of epochs included in the ERP of each condition the same statistical design as in the ERP voltage analyses. None of these tests reached the 0.05 alpha threshold: p = 0.783 for the Group (SI; II) x Stimulus (HIT; CR) mixed ANOVA interaction, and p = 0.905 for the Groups (SI; II) x Sessions (BEG; END) mixed ANOVA interaction on the CR.

### EEG analyses

#### General analysis strategy

To investigate the effect of implementation intentions on inhibitory control and on the effect of practicing the inhibitory control task, we applied a global, data-driven analysis of the EEG data by comparing the ERPs between the groups and experimental conditions for each peri-stimulus time frame and across the whole electrode montage. This approach allowed minimizing biases induced by the a priori selection of a limited number of periods and/or electrodes of interest (for corresponding approaches see e.g. refs 45, 67, 68). Of note, since this approach consists in comparing voltage at each electrode and time point across conditions, it actually includes classical analyses of single electrodes at single time points, which are typically done when focusing on a given ERP component amplitude over a pre-selected set of electrodes. For example, should an effect manifest over the P3 component, we would see a peak in the number of fronto-central electrodes showing a significant effect around 300ms post-stimulus onset^69^.

Once the periods showing modulations in the sensor space were identified, we used them as periods of interest for the statistical analyses of the source estimations. We calculated the sources of the ERP for each subject and each condition previously averaged over the period of interest (i.e. the period showing the ERP modulation). The sources were then statistically compared using the same 2 × 2 designs as for the ERP analyses.

#### Event-related potentials analysis

To assess the immediate effect of II, we computed a Group (SI; II) x Stimulus (HIT; CR) mixed ANOVA at each time-frame of the ERP and for each electrode. To assess the effects of practice, we computed a Group (SI; II) x Session (BEG; END) mixed ANOVA at each time-frame and for each electrode.

This analysis in the sensor space was primarily used to identify when in time the interaction took place; it thus required being sensitive but not necessarily specific. However, we still corrected for spatial and temporal autocorrelation and for multiple test by considering only significant differences on at least 10% of the electrodes and lasting at least 11 contiguous time points at an alpha threshold of 0.05^70^. This threshold was based on a permutation tests assessing the number of continuous significant data points that would be expected to arise by chance (based on 1000 permutations) in temporally auto-correlated noise data. The results of the ERP analyses allowed us to identify periods of interest showing sustained interaction effects over which sources estimations were computed and statistically analyzed.

We focused only on the interaction effects of Group x Stimulus and Group x Session because i) we were interested in how II modulated inhibitory processes depending on the nature of the stimuli or after practice and ii) interaction terms are controlled for group differences unrelated to our experimental questions or other factors potentially confounding the main effects (e.g. the main effect of Session could merely reflect effects of stimulus exposure or fatigue).

As a control analysis, we further compared the Go and NoGo ERPs for the SI and II group separately. This analysis confirmed that our experimental design elicited the classical N2 and P3 components (cf Supplementary Figure 1 for details).

#### Electrical source estimations analysis

For both immediate and practice effects, estimations of electrical sources were analyzed over the time periods identified at the ERP level. Brain sources of ERP modulations were estimated using a distributed linear inverse solution model (a minimum norm inverse solution) combined with the local autoregressive average (LAURA) regularization approach, which describes the spatial gradient across neighboring solution points^71, 72^. LAURA enables investigating multiple simultaneously active sources and selects the configuration of active brain networks which better mimics biophysical behavior of neural fields. In LAURA’s approach, the strength of the potentials at a given location depends on the activity of its neighbor nodes according to electromagnetic laws derived from the quasi-static Maxwell’s equations stating that the strength of a source falls off with the inverse of the squared distance for potential fields (cubic distance for vector fields)^71–73^ (see ref. 74 for a review). LAURA uses a realistic head model, and the solution space consists of 3005 solution points (or ‘nodes’) selected from a 6 × 6 × 6 mm grid equally distributed within the gray matter of the Montreal Neurological Institute’s average brain (MNI, courtesy of Grave de Peralta Menendez and Gonzalez Andino; University Hospital of Geneva, Geneva, Switzerland). The head model and lead field matrix were generated with the Spherical Model with Anatomical Constraints (SMAC)^75^. As an output, LAURA provides current density measures; their scalar values were evaluated at each node. Mostly because of the high indeterminacy of the inverse problem and because the modeling head geometry and spatial conductivity in the forward model is an approximation, source signals reconstruction are only estimations. As a result, the localization of distributed sources based on the LAURA algorithm has limited spatial resolution and may thus fail at differentiating between close structures. However, we computed (conservative) statistical comparisons between our source estimations, which partly limits the influence of spurious activity and ensures the reliability of our results; Given the density of our EEG setup (64 channels), the localization accuracy with LAURA is about 1–2 cm^76^, a resolution compatible with the anatomical labelling of the Automated Anatomical Labeling^77^ resolution we used to interpret our results. Fundamental and clinical studies have confirmed that this level of spatial accuracy can be reached when using this inverse solution approach (see e.g. refs 71, 74, 78–80). The sources estimations were first averaged over the period of interest and the current density at each solution point was subjected to the same [Group (SI; II) x Stimulus (HIT; CR)] or [Group (SI; II) x Session (BEG; END)] design as for the ERPs analyses. A spatial correction for multiple tests was achieved by taking into account only clusters showing a p-value < 0.05 with a spatial-extent criterion (k_E_) of ≥15 contiguous nodes. This spatial criterion was determined using the AlphaSim program (available at http://afni.nimh.nih.gov), assuming a spatial smoothing of 6 mm FWHM and cluster connection radius of 8.5 mm. This program applies a cluster randomization approach; the 10,000 Monte Carlo permutations performed on our lead field matrix revealed a false positive probability of < 0.005 for a cluster greater than 15 nodes (for corresponding approaches see e.g. refs 81–83).

### Data availability

The data that support the findings of this study are available from the corresponding author upon reasonable request.

## Electronic supplementary material

Supplementary material
