# Supplementary material for: Spatiotemporal brain dynamics supporting the immediate automatization of inhibitory control by implementation intentions
Source: Sci Rep. 2017 Sep 7;7:10821. doi: 10.1038/s41598-017-10832-x (PMC5589860; doi:10.1038/s41598-017-10832-x)
Supplement: Supplementary file 1 — Supplementary material [file 41598_2017_10832_MOESM1_ESM.doc]

**Spatiotemporal brain dynamics supporting the immediate automatization of inhibitory control by implementation intentions**

Michael De Pretto, Lucien Rochat, Lucas Spierer

**Supplementary information**

**Supplementary Figure 1**


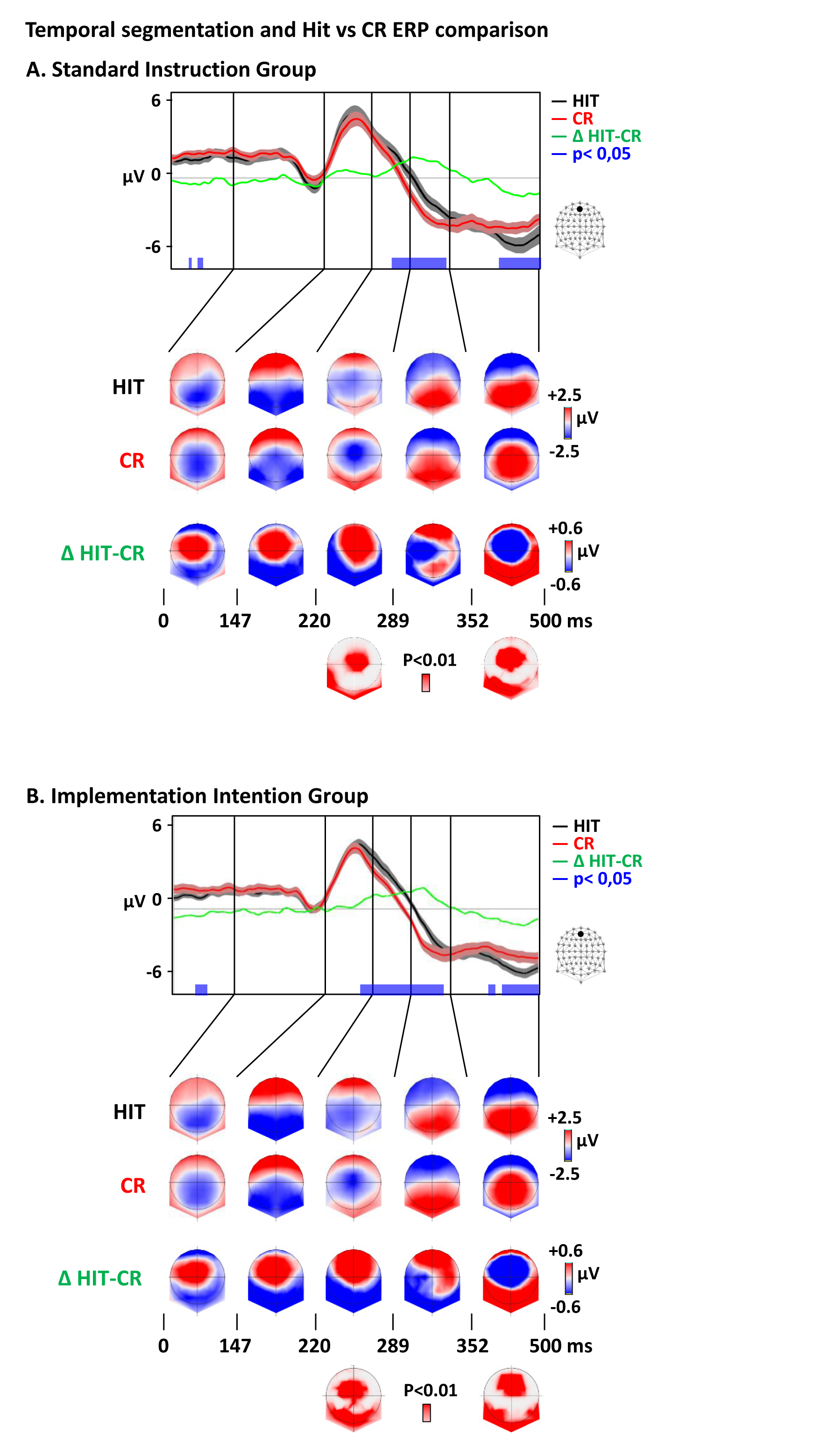


**Supplementary Figure 1:**

In order to examine if our experimental paradigm successfully elicited the typical N2 and P3 inhibition-related ERP components, we submitted the group-averaged ERP data to a hierarchical clustering based on an atomize and agglomerate approach (Brunet 2011; Murray 2008; Michel 2009). This approach is based on evidence that the ERP map topography does not vary randomly across time, but remains quasi-stable over 20-100 msec functional microstates -i.e. the ERP components- before rapidly switching to other stable periods (Lehmann and Skrandies, 1980; Pascual-Marqui 1995; Cacioppo 2014). As in previous literature based on this approach (e.g. Fargier 2016; Maitre 2017; Laganaro 2012), the optimal number of clusters that explained the best the grand-average data sets across conditions was identified using a modified version of the cross validation criterion combining a cross-validation criterion and the Krzanovski-Lai criterion (Tibshirani et al., 2005; see Murray 2008). This analysis enabled identifying the series of ERP component in our data in a data-driven manner. The clustering analysis revealed 5 time windows comprising distinct ERP components (0-147 ms; 148-220 ms; 221-289 ms; 290-352 ms; 353-500 ms). The timing and the topography of these components corresponded to the typical sequence of components observed in visual Go/NoGo tasks. We report the ERP topographies of the identified ERP components for the Go (Hit) and NoGo (Correct rejection) for each of these components and for the SI and II groups separately, as well as the topography of the difference between the components in the Hit-CR condition. We also report the group-averaged waveforms at an exemplar electrode within the cluster showing the significant interaction and the t- and p- values of the Hit vs CR t-tests over time.

This analysis confirms the presence of a N2 and P3 components (with negative fronto-central topography ca. 220 -330ms and a positive central topography 330-500ms, respectively, cf e.g. Falkenstein 1999), and that our interaction effect takes place at the beginning of the N2 component (i.e. during a period of stable topography).

**References:**

Brunet, D., Murray, M. M., & Michel, C. M. (2011). Spatiotemporal analysis of multichannel EEG: CARTOOL. Computational Intelligence and Neuroscience, 2011, 813870. https://doi.org/10.1155/2011/813870

Cacioppo, S., Weiss, R. M., Runesha, H. B., & Cacioppo, J. T. (2014a). Dynamic spatiotemporal brain analyses using high performance electrical neuroimaging: Theoretical framework and validation. Journal of Neuroscience Methods, 238, 11–34. https://doi.org/10.1016/j.jneumeth.2014.09.009

Cacioppo, S., Weiss, R. M., Runesha, H. B., & Cacioppo, J. T. (2014b). Dynamic spatiotemporal brain analyses using high performance electrical neuroimaging: theoretical framework and validation. Journal of Neuroscience Methods, 238, 11–34. https://doi.org/10.1016/j.jneumeth.2014.09.009

Falkenstein, M., Hoormann, J., & Hohnsbein, J. (1999). ERP components in Go/Nogo tasks and their relation to inhibition. Acta Psychologica, 101(2–3), 267–91. Retrieved from http://www.ncbi.nlm.nih.gov/pubmed/10344188

Fargier, R., & Laganaro, M. (2016). Neurophysiological Modulations of Non-Verbal and Verbal Dual-Tasks Interference during Word Planning. PloS One, 11(12), e0168358. https://doi.org/10.1371/journal.pone.0168358

Laganaro, M., Valente, A., & Perret, C. (2012). Time course of word production in fast and slow speakers: a high density ERP topographic study. NeuroImage, 59(4), 3881–8. https://doi.org/10.1016/j.neuroimage.2011.10.082

Lehmann, D., & Skrandies, W. (1980). Reference-free identification of components of checkerboard-evoked multichannel potential fields. Electroencephalography and Clinical Neurophysiology, 48(6), 609–21. Retrieved from http://www.ncbi.nlm.nih.gov/pubmed/6155251

Maitre, N. L., Key, A. P., Chorna, O. D., Slaughter, J. C., Matusz, P. J., Wallace, M. T., & Murray, M. M. (2017). The Dual Nature of Early-Life Experience on Somatosensory Processing in the Human Infant Brain. Current Biology : CB, 27(7), 1048–1054. https://doi.org/10.1016/j.cub.2017.02.036

Murray, M. M., Brunet, D., & Michel, C. M. (2008). Topographic ERP analyses: a step-by-step tutorial review. Brain Topography, 20(4), 249–64. https://doi.org/10.1007/s10548-008-0054-5

Pascual-Marqui, R. D., Michel, C. M., & Lehmann, D. (1995). Segmentation of brain electrical activity into microstates: model estimation and validation. IEEE Transactions on Bio-Medical Engineering, 42(7), 658–65. https://doi.org/10.1109/10.391164

**Supplementary Figure 2 : Experimental design : Stimuli across blocks**

| **Block “A”** | | **Block “E”** | | **Block “O”** | | **Block “blue”** | | **Block “green”** | | **Block “red”** | |
| --- | --- | --- | --- | --- | --- | --- | --- | --- | --- | --- | --- |
| **Go** | **NoGo** | **Go** | **NoGo** | **Go** | **NoGo** | **Go** | **NoGo** | **Go** | **NoGo** | **Go** | **NoGo** |
| **E** | **A** | **A** | **E** | **A** | **O** | **A** | **A** | **A** | **A** | **A** | **A** |
| **E** | **A** | **A** | **E** | **A** | **O** | **E** | **E** | **E** | **E** | **E** | **E** |
| **E** | **A** | **A** | **E** | **A** | **O** | **O** | **O** | **O** | **O** | **O** | **O** |
| **O** |  | **O** |  | **E** |  | **A** |  | **A** |  | **A** |  |
| **O** |  | **O** |  | **E** |  | **E** |  | **E** |  | **E** |  |
| **O** |  | **O** |  | **E** |  | **O** |  | **O** |  | **O** |  |

**Supplementary figure 2**

Table illustrating the six types of block and their stimuli. “Block “A”” for example means that the NoGo was the A letter irrespective of the color. The blocks were presented in a randomized order to the participants during the session. Each block was presented two times in a row, and then two different blocks were presented etc.
